# Detection of epileptic seizures through EEG signals using entropy features and ensemble learning

**DOI:** 10.3389/fnhum.2022.1084061

**Published:** 2023-02-01

**Authors:** Mahshid Dastgoshadeh, Zahra Rabiei

**Affiliations:** Department of Engineering, Aliabad Katoul Branch, Islamic Azad University, Aliabad Katoul, Iran

**Keywords:** epileptic seizures, machine learning, ensemble learning, entropy features, discrete wavelet transform, ANOVA, FSFS

## Abstract

**Introduction:**

Epilepsy is a disorder of the central nervous system that is often accompanied by recurrent seizures. World health organization (WHO) estimated that more than 50 million people worldwide suffer from epilepsy. Although electroencephalogram (EEG) signals contain vital physiological and pathological information of brain and they are a prominent medical tool for detecting epileptic seizures, visual interpretation of such tools is time-consuming. Since early diagnosis of epilepsy is essential to control seizures, we present a new method using data mining and machine learning techniques to diagnose epileptic seizures automatically.

**Methods:**

The proposed detection system consists of three main steps: In the first step, the input signals are pre-processed by discrete wavelet transform (DWT) and sub-bands containing useful information are extracted. In the second step, the features of each sub-band are extracted by approximate entropy (ApEn) and sample entropy (SampEn) and then these features are ranked by ANOVA test. Finally, feature selection is done by the FSFS technique. In the third step, three algorithms are used to classify seizures: Least squared support vector machine (LS-SVM), K nearest neighbors (KNN) and Naive Bayes model (NB).

**Results and discussion:**

The average accuracy for both LS-SVM and NB was 98% and it was 94.5% for KNN, while the results show that the proposed method can detect epileptic seizures with an average accuracy of 99.5%, 99.01% of sensitivity and 100% of specificity which show an improvement over most similar methods and can be used as an effective tool in diagnosing this complication.

## 1. Introduction

Epilepsy is a chronic neurological disorder of the central nervous system that occurs in the brain and usually occurs as sudden and recurrent seizures (Siddiqui et al., 2020). This disease is known as the third most common neurological disorder after stroke and Alzheimer’s (Sharma et al., 2020). During epileptic seizures, the normal pattern of nervous system activity is disrupted, leading to impaired movement, losing control of bowel or bladder function, and loss of consciousness. As a result, epileptic seizures can increase the risk of paralysis, fractures, and even sudden death in patients (Natu et al., 2022). It is estimated that more than 70% of patients with epilepsy, if diagnosed and treated promptly, can enjoy a normal life without seizures (Cherian and Kanaga, 2022). Thus, if epilepsy would be diagnosed early, then we will be able to prevent its unintended consequences.

Today, Electroencephalography (EEG) is widely used to monitor the electrical function of brain neurons. This method is a valuable tool for assessing and diagnosing epilepsy. At present, the general method for diagnosing epileptic seizures is mainly based on visual examination of a large amount of information in EEG signals by physicians, which is usually time consuming and the probability of a mistake is high. For this reason, it is important to provide an automated method for processing EEG signals and detecting epileptic seizures. However, EEG signals are highly complex, nonlinear, and non-stationary in nature due to the complexity of the connections between the billions of neurons in the brain (Daftari et al., 2022). In this regard, how to effectively diagnose epileptic seizures using EEG signals is a major challenge.

The amount of research done to diagnose epileptic seizures through EEG signals using machine learning techniques is very extensive and every year we see many new approaches to solve this problem.

Electroencephalography and functional magnetic resonance imaging (fMRI) are the most vital tools for studying brain activity but both of them are very sensitive to synaptic activity, some researchers used simultaneous EEG and fMRI measurements and proved that simultaneous recording of EEG and fMRI can help us to better understand the physiological brain networks and the correspondence between the brain hemodynamic signal and electrical neural activity (Ebrahimzadeh et al., 2022). The simultaneous EEG–fMRI is a non-invasive method which is significant for those patients who are pharmacoresistant and surgical candidates and it can be used to find the pathophysiological mechanisms of the discharges (Sadjadi et al., 2021). In Sadjadi et al. (2022), first EEG-fMRI data from 18 patients with focal epilepsy recorded and then two approaches for analyzing fMRI data alone containing spatial independent component analysis (sICA) and functional connectivity (FC) applied to data. Ebrahimzadeh et al. (2021b) extracted the epilepsy-related components with an ICA analysis and then they prioritized these elements according to the cross-correlation between the spike-template and the time-series of each component and their alignment with the complementary physiological information. Also, they convolved the time series with the hemodynamic response function (HRF) to produce a regressor for a general linear model (GLM) analysis.

In Chen et al. (2019) a solution for the diagnosis of epileptic seizures using nonlinear dynamic features is presented. In this method, pre-processing and noise removal operations are performed using discrete wavelet transform (DWT). Nonlinear dynamic features are then used to describe EEG signals. Finally, a SVM is used to classify features and diagnose epileptic seizures. It should be noted that our proposed solution, is an attempt to continue this research and to improve its performance using the ensemble learning.

In Wang et al. (2017), a framework for the automatic diagnosis of epilepsy is proposed based on a combination of multi-domain features and nonlinear analysis of EEG signals. In the first step, the EEG signals are pre-processed using wavelet analysis to eliminate noise in the signal. Then a set of candidate features in the time, frequency, and time-frequency domains, in addition to nonlinear features based on information theory are extracted. These features are extracted from the five frequency bands of the signal and then their dimensions are reduced using Principal component analysis (PCA) and variance analysis. Finally, the optimal combination of extracting features is evaluated through different classifications. The results show that the highest detection accuracy can be achieved by using SVM.

In Jaiswal and Banka (2017), a feature extraction method based on the conversion of local patterns to detect epileptic seizures through EEG signals is presented. This method introduces two feature extraction techniques for epileptic EEG signal classification. Both of these techniques local neighbor descriptive pattern (LNDP) and One-dimensional local gradient pattern (1DLGP) are based on local pattern conversion, which uses histogram-based features to increase execution speed and improve classification accuracy. Both techniques work better than local binary patterns (LBP) in term of maintaining the structural features of the patterns. In this study, the application of various learning models in diagnosis has been studied, which shows the superiority of artificial neural network (ANN) over other learning models.

In Swami et al. (2016), an automated system for classifying EEG signals and detecting epileptic seizures has been presented. In this method, the input signals are first decomposed using wavelet transform into time-frequency bands up to the sixth level. In this method, all the detail bands and the last approximation coefficients are used to extract the signal features. The extracted feature set of each EEG includes: energy, standard deviation, root mean square, Shannon entropy, mean, and maximum values of the signal. Finally, this set of features is classified through a general regression neural network (GRNN). The method presented in Sarić et al. (2020), uses a multi-layer perceptron (MLP) based on field programmable gate array (FPGA) to classify the types of epileptic seizures. In this method, a low-pass filter is used to pre-process EEGs and remove noise from them. Continuous wavelet transform (CWT) has also been used to extract signal features. In Hassan et al. (2020) The Boosting technique is used to diagnose epileptic seizures. In Boosting technique, a combination of several identical learning models formed with different training samples; is used to build a classification model. This research shows that the use of multiple learning models can be effective in increasing the accuracy of diagnosis.

In a recent study by Zhou et al. (2018), EEG signals from 21 patients recorded and six contacts were selected for each patient by visual inspection. Also, they used the other database from CHB-MIT. In that method, a convolutional neural network (CNN) used to distinguish ictal, preictal, and interictal phases. Time or frequency domain signals are used as inputs for classification. They did three different types of experiments and they concluded that frequency domain signals performed better than time domain signals.

Moreover, Singh and Malhotra (2022) have presented a cloud-fog integrated smart neurocare approach. They selected a single channel from multichannel EEG signals and after filtering, it segmented into short-duration segments. In this approach, three deep learning algorithms include stacked autoencoder (SA), recurrent neural network (RNN) and CNN are used to classify and finally they concluded that CNN model worked better than other classifiers.

In a method proposed by Gao et al. (2022) epileptic EEG signals transformed to power spectrum density energy diagrams (PSDEDs) and deep convolutional neural networks (DCNNs) used to extract features. Shoeibi et al. (2021) used the tunable Q-factor wavelet transform (TQWT) to decompose EEG signals to sub-bands and then fuzzy entropy calculated from different sub-bands. Then, the standard adaptive neuro-fuzzy inference system (ANFIS) and other methods like ANFIS-GOA, ANFIS-PSO, and ANFIS-BS used for classification.

Other researchers suggested multidomain features ranging from the time domain to the frequency domain and compared ML and DL approaches for detecting epileptic seizures. In machine learning approach, after filtering EEG signals, feature extraction applied with DWT and MODWT. Then they used some classifiers such as SVM kernels, K-nearest neighbors (KNN), Naive Bayes (NB), and artificial neural networks. In the other approach, they used two deep learning algorithms contain CNN and RNN for automated feature extraction (Daftari et al., 2022).

Ebrahimzadeh et al. (2021a), introduced a component-based EEG-fMRI method for Localizing confined epileptic foci in patients with presumed multifocality or unclear focus. In this research, the neural behavior of epileptic generators is identified and used as the input of a generalized linear model. This study was a continuation of the research previously conducted by Ebrahimzadeh et al. (2019). Rahimpour et al. (2020), conducted a functional near-infrared spectroscopy (fNIRS) validation study on tracking differential activation of primary and supplementary motor cortex across timing tasks. fNIRS, can be considered as a replacement for fMRI method, in order to examine changes in cortical hemodynamics. Table 1 summarize the characteristics of our proposed method and some similar studies.

**TABLE 1:** Characteristics of recent methods.

References	Method	Data
Wang et al., 2017	Combination of multi-domain features and nonlinear analysis.	EEG signal
Jaiswal and Banka, 2017	Feature extraction method based on the conversion of local patterns.	EEG signal
Sarić et al., 2020	Multi-layer perceptron (MLP) based on field programmable gate array (FPGA)	EEG signal
Hassan et al., 2020	Boosting technique (combination of several identical learning models formed with different training samples).	EEG signal
Zhou et al., 2018	Convolutional neural network (CNN) based on raw EEG signals used to distinguish different segments of epileptic seizures	EEG signal
Singh and Malhotra, 2022	A cloud-fog integrated smart neurocare and deep learning used to detect the occurrence of epileptic seizures.	EEG signal
Shoeibi et al., 2021; Daftari et al., 2022	The tunable Q-factor wavelet transform (TQWT) used to decompose EEG signals and the standard adaptive neuro-fuzzy inference system (ANFIS), ANFIS-GOA, ANFIS-PSO, and ANFIS-BS used for classification.	EEG signal
Sadjadi et al., 2022	Spatial independent component analysis (sICA) and functional connectivity (FC) analysis.	fMRI
Ebrahimzadeh et al., 2021b	ICA analysis used to extract epilepsy components and cross-correlation prioritized the elements. HRF used to produce a regressor for GLM analysis.	fMRI and EEG signal
Proposed method	ApEn and SampEn extracted from EEG signal, FSFS used to select features and LS-SVM, KNN and NB used to classify.	EEG signal

In this paper, a new method for diagnosing epileptic seizures using data mining and machine learning techniques is presented. The contribution of this paper is 2-fold: First, the extraction of entropy-based features from the wavelet coefficients of EEG signals can play an effective role in increasing the accuracy of seizure detection, which is studied in the proposed method. Second, the combination of several learning models for feature classification can be effective in reducing detection error. This strategy is called ensemble learning and its efficiency in the diagnosis of epileptic seizures will be discussed in this paper.

These two cases, which can be considered as innovative aspects of the current research, distinguish the proposed method from the previous solutions. The remainder of this paper is organized as follows: In section “2 Proposed method” for automatic diagnosis of epileptic seizures is described in details. In section “3 Results” are presented. Finally, in section “4 Conclusion” are discussed and some suggestions for further research are provided.

## 2. Proposed method

The proposed method in this paper, diagnoses epileptic seizures in EEG signals through the following processing steps:

1. Preprocessing
2. Features extraction and features selection
3. Classification

**FIGURE 1:**
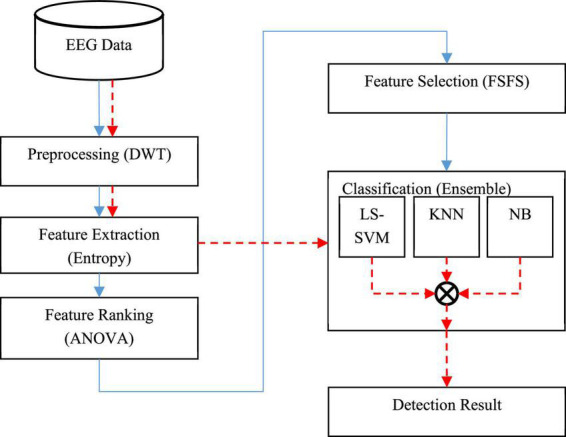
Block diagram of the proposed method.

The process of diagnosing the disorder using the proposed method is shown as a block diagram in Figure 1.

In Figure 1, the sequence of the operations related to the training phase is shown as continuous line; while the sequence of the test phase (diagnosing through new samples) is shown as a dashed line. In the training phase, a set of EEG data is first used to build learning models. In this way, input signals are first preprocessed to eliminate noise and wavelet sub-bands of signals containing useful information related to epileptic seizures are extracted. In the next step, two entropy-related features, namely approximate entropy (ApEn) and sample entropy (SampEn), are used to describe EEG features. Then, the analysis of variance (ANOVA) test will be used to rank the extracted features, and finally, an optimal subset of the features will be selected using the forward sequential feature selection technique (FSFS). In the next step, three machine learning models, namely: least squared support vector machine (LS-SVM), KNN, and NB will be used to train and build the learning model. The result of this phase will be three trained learning models for classifying new samples.

After the implementation of the training phase, the obtained learning models can be used in the test phase. Thus, in the test phase of the proposed model, the preprocessing and feature extraction processes are performed on new samples and the resulting features are used as input to train learning models. Finally, the output of the diagnostic system based on the presence or absence of epileptic seizures is generated using the voting technique. The details of each of these steps will be discussed below.

### 2.1. Preprocessing

The purpose of data preprocessing is to prepare the input EEG signals for proper processing in the next steps. For this purpose, the destructive effect of noise on EEG signals must be eliminated. Many signals, such as EEG, have transient properties. In such cases, the Fourier transform cannot be applied directly to the signal. For this reason, in recent years, various methods for removing noise from signals have been studied. In this research, DWT is used to remove noise from EEG signals. DWT includes a wide range of basic functions. These functions provide different options for processing EEG signals in different conditions that can be matched to the characteristics of EEG signals in epileptic seizures (Chen et al., 2017).

**FIGURE 2:**
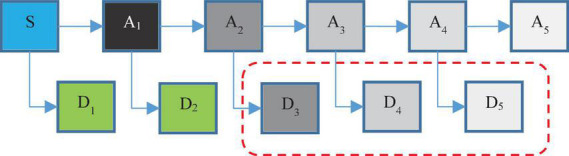
The hierarchical process of discrete wavelet transform (DWT) in proposed method.

Using discrete wavelet analysis, single-channel EEG signals can be decomposed into several sub-bands. Then, through these decomposition signals, more features can be extracted, thereby improving the accuracy of epilepsy diagnosis. Each segment of the decomposed signal can be thought of as a wavelet coefficient and a scale factor. Wavelet coefficients and scaling coefficients are obtained in each step of applying the wavelet filter and scaling filter to the main time series, which is repeated in the form of the pyramidal algorithm shown in Figure 2. In this figure, S represents the primary EEG signal. A_*i*_ indicates low frequency data in *i*-th level and D_*i*_ indicates high frequency data in this level.

In fact, in wavelet analysis, the data are divided into two series of high frequency and low frequency data. The small amount of data obtained by applying the father wavelet to the main series indicates the main features of the series. High-frequency data are also obtained by applying the mother wavelet on the main series, which is often called noise, and with further progress in the decomposition levels, the probability of noise in high-frequency data is reduced (Chen et al., 2017). For example, in Figure 2, the probability of noise in A_1_ data is very high; while the probability of noise in A_5_ data will be very low. In fact, the main purpose of wavelet decomposition is to separate the main features from noise.

**FIGURE 3:**
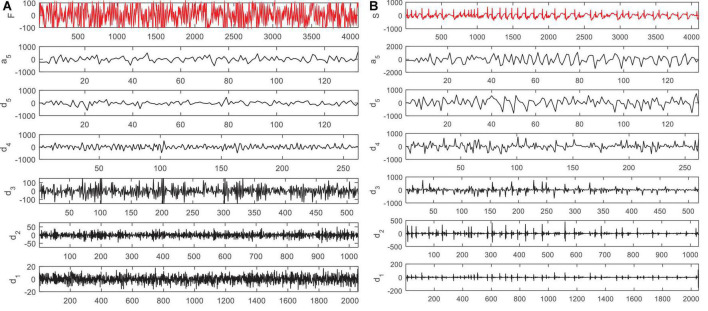
Sub-bands resulting from decomposition of an electroencephalogram (EEG) signal in panel **(A)** F dataset and **(B)** S dataset by discrete wavelet transform (DWT).

According to Figure 2, in the proposed method, EEG signals are decomposed into six sub-bands (*a*_5_, *d*_1_, *d*_2_, *d*_3_, *d*_4_, *d*_5_). Seizures consist of three stages: beginning, middle (ictal), and end, and the period between seizures is called interictal. For this purpose, two datasets S (ictal EEG) and F (interictal EEG) will be used in the EEG signal database. Also, the wavelet function used to decompose EEG signals by DWT is the Daubeches-4 (DB4) function. Figure 3 shows the sub-bands resulting from the application of DWT based on the DB4 function.

The conversion of the S and F signal domains from time to frequency indicates that the frequency range of the sub-bands extracted from the decomposition of the EEG signals are as follows:

1. The residual signal a_5_ with the frequency range of 0–3 Hz
2. d_1_ with the frequency range of 50–100 Hz
3. d_2_ with the frequency range of 25–50 Hz
4. d_3_ with the frequency range of 12–25 Hz
5. d_4_ with the frequency range of 6–12 Hz
6. d_5_ with the frequency range of 3–6 Hz

The frequency of epileptic EEG signals is usually in the range of 3–25 Hz. The frequency of d_1_ and d_2_ signals is out of the frequency range of EEG signals during epileptic seizures. On the other hand, it has been shown in Chen et al. (2019) that 3, 4, and 5 signal channels can well describe the characteristics of EEG signals during seizures. Therefore, in the proposed method, we will use the same channels (i.e., d_3_, d_4_, and d_5_ sub-bands) to calculate entropy features.

### 2.2. Feature extraction and feature selection

In the second step of the proposed method, features are extracted from EEG signals. Choosing the right set of features for describing the patient attributes, is a key process in disease diagnosis systems (Jin et al., 2022). It is important to avoid selecting irrelevant features, as well as selecting the most related ones to the disease (Yang et al., 2022). For this purpose, two entropy-based criteria will be used to derive the features, which will be described in this section.

#### 2.2.1. Entropy-based feature extraction

Entropy has roots in physics and it is a measure of disorder, or unpredictability, in a system. Applying the concept of entropy to time series like EEG is a way to quantify, in a statistical sense, the amount of uncertainty or randomness in the pattern, which is also roughly equivalent to the amount of information contained in the signal. It is a set of nonlinear criteria used to indicate the complexity and random nature of time series. In the proposed model, these nonlinear criteria are used to describe the dynamics of EEG signals, taking into account the nature of their instability and nonlinearity. For this purpose, two entropy-based features are extracted from each sub-band (d_3_, d_4_, and d_5_) of the EEG signal. These two features are: ApEn and SampEn. Both of them are very sensitive to input parameter choice and SampEn is a modification of ApEn. So, we chose these two features to show the SampEn priorities.

##### 2.2.1.1. Approximate entropy

Approximate entropy can descsribe complex signals based on the characteristics of certainty, turbulence, or randomness. The ApEn criterion indicates the probability that similar patterns are not followed by observations made by subsequent similar observations. A high ApEn value indicates high irregularity in the series, while a low ApEn value indicates a time series with multiple repetitive patterns. Having a time series such as *X* = {*x*_*i*_, *i* = 1, 2, …, *N*}, where *N* is the length of the time series, ApEn is computed using several consecutive steps. For this purpose, first a sequence of delayed vectors with the dimensionality of *m* is constructed as follows (Srinivasan et al., 2007):


(1) $$x_{m}\left(i\right)=\left\{x_{i},x_{i+t},x_{i+2t},\ldots ,x_{i+\left(m-1\right)t}\right\}\in {\mathbb{R}}^{m},1\leq i\leq N-\left(m-1\right)t$$

Where, *m* and *t* are both positives integers that show the vector and time delay, respectively. For each *i*, in the interval 1 ≤ *i* ≤ *N* − (*m* − 1)*t*, the full correlation relation $C_{i}^{m}\left(r\right)$ will be as follows (Srinivasan et al., 2007):


(2) $$C_{i}^{m}\left(r\right)=\frac{1}{N-\left(m-1\right)t}\sum_{j=1}\theta \left(r-d\left(x_{m}\left(i\right),x_{m}\left(j\right)\right)\right)$$

Where, *r* is tolerance parameter and *d*(*x*_*m*_(*i*), *x*_*m*_(*j*)) is the distance calculation function and is calculated as follows (Srinivasan et al., 2007):


(3) $$d\left(x_{m}\left(i\right),x_{m}\left(j\right)\right)={\text{max}}_{\text{k}=1,2,\ldots ,m}\left(\left|x_{i+\left(k-1\right)t}-x_{j+\left(k-1\right)t}\right|\right)$$

The function θ(.) is also defined as follows (Srinivasan et al., 2007):


(4) $$\theta \left(x\right)=\left\{ \begin{array}{c} 1\;ifx>0\\ 0\;ifx\leq 0 \end{array}\right.$$

In the next step, we define the function ∅*^m^*(*r*) as follows (Srinivasan et al., 2007):


(5) $$\emptyset^{m}\left(r\right)=\frac{1}{N-\left(m-1\right)t}\sum_{i=1}^{N-\left(m-1\right)t}\log C_{i}^{m}\left(r\right)$$

Finally, for the constant values of *m, r* and *t*, ApEn will be calculated by the following equation (Srinivasan et al., 2007):


(6) $$ApEn\left(m,r,t,N\right)=\emptyset^{m}\left(r\right)-\emptyset^{m+1}\left(r\right)$$

Typically, *m* is chosen as 2 or 3, and *r* highly depends to the problem. In the proposed method, *m* = 2, *r* = 0.2 is equal to the standard deviation of EEGs and *t* = 1.

##### 2.2.1.2. Sample entropy

Sample entropy, or SampEn, is a modified version of ApEn and is used to evaluate the complexity of physiological time series signals and to diagnose disease states. The SampEn criterion has two main advantages over the ApEn method: independence from data length and simple implementation. This criterion is calculated as follows (Richman et al., 2004):


(7) $$SampEn=-\text{log}\left(\frac{A}{B}\right)$$

Where, *A* is the total number of pairs in the vector *x*_*m*_(*i*) (Eq. 1), which for the length *m*+1, their distance is less than *r*; and *B* is the total number of pairs in this vector which for the length of *m*, their distance is less than *r*. In this research, we consider the value of *m* to be 2.

After applying the DWT on the input signal, the signals obtained from the wavelet coefficients of channels 3, 4, and 5 (d_3_, d_4_, and d_5_) are used to calculate the entropy features and for each sample, in total 6 entropy features (3 × 2) are obtained. In the next step, these entropy features will be used to rank and select the optimal feature set.

#### 2.2.2. Ranking and selecting features

Feature selection is a vital step in machine learning method which can improve the performance of later data processing. Vaghela et al. (2014) original EEG signals usually have a lot of irrelevant and redundant information which we do not need them in our experiments. With feature selection, we can reduce the number of irrelevant features and increase the accuracy of process. Also, it increases the prediction power of the algorithms by selecting the most critical variables. In the previous step, six features were extracted using two entropy algorithms: ApEn and SampEn, based on three EEG sub-band signals. This feature set may contain a number of unrelated features. Therefore, in the proposed method, one-way ANOVA test will be used to rank the importance of these features and select the most related ones to epileptic seizures. The *F*-statistics of the features are calculated using the ANOVA test. In general, features with higher values of *F*-statistics are more important in the classification phase and are therefore more significant. After ranking the features, the feature selection operation will be done using FSFS method. FSFS is mostly used to select the most appropriate and significant subset of ranked features. This algorithm forms a subset of optimal features by selecting six features in a row and based on their ranking. By adding each new feature to the feature subset at each step, the learning model classification error rate is checked. When adding a new attribute reduces the performance of the classification model, the process of adding the feature is terminated and the currently selected attribute subset is considered as the optimal subset. After selecting the optimal features, the feature classification operation will be performed, which we will discuss in the next section.

### 2.3. Feature classification by ensemble learning

The last step of the proposed method is to classify the selected features. In the proposed method, this operation is performed using the ensemble learning. Ensemble learning is an efficient mechanism in improving the accuracy of collaborative classifiers, compared to the case where each classifier is used separately (Ranjbari et al., 2021). The algorithms used to classify the features and detect epileptic seizures are: LS-SVM, NB, and KNN. In the following, we will describe the process of classification by these algorithms.

#### 2.3.1. Least squares-support vector machine

The first learning model used in the proposed ensemble model is the SVM. SVM can be described by two hyper-planes with a definite boundary and position relative to each other. Each hyper-plane belongs to one of the target classes and the smallest distance between the samples of each class is considered as the margin of the hyper-plane. The SVM is a classification algorithm that tries to maximize the classification accuracy by maximizing the margin between the sample holding pages of each class. To find the line separating the classes, these algorithms start from two parallel lines and move these lines in opposite directions so that each line reaches a sample of a specific category on its side. After this step, a border is formed between two parallel lines. The wider the bar, the more the algorithm is able to maximize the margin, and the goal is to maximize the margin (Pisner and Schnyer, 2020). In the proposed method, a SVM with least squares is used to classify the features and minimize the classification error. Since, classic SVM has high computational burden. LS-SVM in this proposed method uses the linear kernel function to classify databases and diagnose epileptic seizures. Suppose P is a sample database as $\left({\vec{x}}_{1},y_{1}\right),\ldots ,\left({\vec{x}}_{P},y_{P}\right)$ and ${\vec{x}}_{i}=\left\{a_{1},a_{2},\ldots ,a_{K}\right\}$ is vector characteristics of each instance and *y*_*i*_ ∈ {−1, 1} indicates that each sample belongs to one of two target categories. The purpose is to create hyperplane with the most margins so that classes can be separated. In other words, the hyperplane should be specified for each instance ${\vec{x}}_{i}$, the distance between the sample and the hyper-page is the maximum. Each hyperplane can be described as follows (Han et al., 2011):


(8) $$\vec{w}.\vec{x}=b$$

In the above equation, $\vec{w}$ is normal hyperplane vector and b is the margin factor. For a support vector machine with a linear core, two parallel hyperplane can be specified as the distance between the sample and the margin of the hyperplanes reach the maximum. These hyperplanes for support vector machine with a linear core and two target classes are described as $\vec{w}.\vec{x}-b=1$ and $\vec{w}.\vec{x}-b=-1$. In terms of numeral, the distance between these two hyperplanes is equal to $\frac{2}{||\vec{w}||}$. So, for maximizing the distance between hyperplanes $||\vec{w}||$ should be minimized. Therefore, the following terms are established for each sample (Han et al., 2011):


(9) $$\left\{ \begin{array}{c} \vec{w}.\vec{x_{i}}-b\geq 1,ify_{i}=1\\ \vec{w}.\vec{x_{i}}-b\geq -1,ify_{i}=-1 \end{array}\right.$$

Thus, classification of samples is turned to an optimization question like the following equation (Han et al., 2011):


(10) $$\min\left(||\vec{w}||\right)|y_{i}\left(\vec{w}.\vec{x_{i}}-b\right)\geq 1,1\leq i\leq P$$

The purpose of the core function in support vector machine is to find optimal values for $\vec{w}$ and b in Eq. 10. In questions which data are not dissociable as linear, with using nonlinear cores, we map the data to a space with more dimensions to separate them linearly in this new space.

#### 2.3.2. Naive Bayes

The second learning model used in the proposed ensemble system is NB. The Bayesian method is simply a method of classifying phenomena based on the probability of its occurrence. In the NB classification, the probability of an event occurring in the future can be inferred from previous events. Bayesian classification is used for problems in which each instance of *x* is selected by a set of attribute values and the objective function *f(x)* from a set such as *V*. The Bayesian mechanism for classifying a new sample is to identify the most likely class or target value of *v*_*MAP*_ by having the attribute values *a*1, *a*_2_, …, *a*_*n*_ that describe the sample. In the proposed method, a simple Bayesian mechanism based on Yang (2018) is used to classify the features.

#### 2.3.3. K-nearest neighbor

The KNN method is one of the simplest machine learning algorithms for classification. In this algorithm, a sample is categorized by a majority vote of its neighbors, and this sample is determined in the most general class among k closest neighbors. The KNN method is applicable to many methods because it is effective, non-parametric, and easy to implement. For this reason, in the proposed method, it is considered as one of the ensemble model algorithms. This algorithm classifies a test sample based on its k closest neighbors. A point in space belongs to a class in which most of the instructional points belong to that class within the instance closest to K (Peterson, 2009). In the proposed method, the Euclidean distance criterion is used in the KNN model. Also, the parameter K or the number of close neighbors is set equal to 3.

The last step in diagnosing epileptic seizures in the proposed method is to use the voting technique. The purpose of the voting technique is to improve the accuracy of the classification algorithms, compared to the case where each algorithm is used separately. Each of the classification algorithms may have an error in classifying some samples; therefore, the purpose of voting techniques is to reduce the resulting error and increase the accuracy of classification. Accordingly, in the last step of the proposed method, the LS-SVM, NB and KNN classification models perform the classification operations of the test samples separately; and finally, by voting the results of all three models, the final output of the system is determined.

## 3. Results

In this section “2 Proposed method” is implemented using MATLAB software and the performance of the proposed method is examined. The efficiency of the proposed method is also compared with other learning models. The database used in the experiments, is the epileptic seizures EEG data prepared by Bonn University. This database consists of five datasets (Z, N, O, F, and S), each set of which contains 100 EEG signal samples and it is shown in Table 2. In this research, two data sets F and S including 200 samples of single channel EEG signals have been used. The samples of these two data sets are divided into two categories: normal (100 samples) and containing epileptic seizures (100 samples). The data were recorded at a sampling rate of 173.61 Hz and the total length of each signal was approximately 23.6 s (University of Bonn, 2021).

**TABLE 2:** Dataset from BONN university.

Dataset	Number of samples	Patients	Phase	Setup
S	100	Epilepsy	Seizure	Intracranial EEG
F	100	Epilepsy	Interictal	Intracranial EEG
O	100	Healthy	Closed eyes	Surface EEG
N	100	Epilepsy	Interictal	Intracranial EEG
Z	100	Healthy	Open eyes	Surface EEG

**FIGURE 4:**
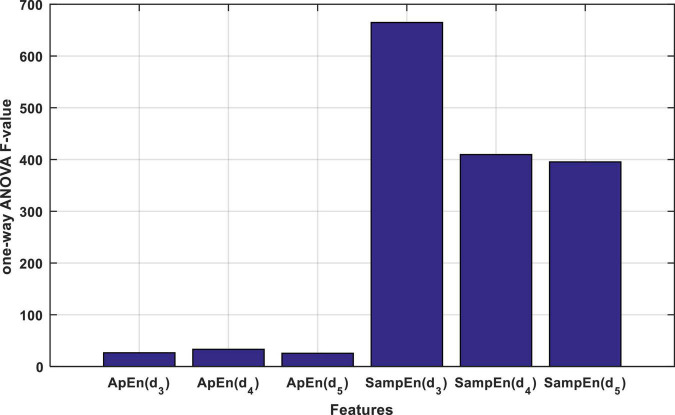
The result of calculating the *F* statistic for each entropy property by one-way ANOVA test.

As described in the previous section, each input signal is described through the approximate and SampEn criteria for each of the d3, d4, and d5 wavelet sub-bands. Thus, each input signal will be described with 6 entropy values, and thus, the entire database will be stored as a matrix with dimensions of 6 × 200. In the proposed method, one-way ANOVA test and *F*-statistic were used to rank entropy features. The results of this ranking are shown in Figure 4.

**FIGURE 5:**
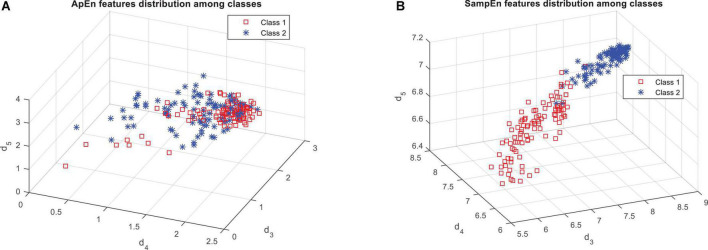
Distribution of samples on target classes based on the features obtained from panel **(A)** approximate entropy (ApEn), and **(B)** sample entropy (SampEn).

Figure 4 shows that the features based on the SampEn, significantly have a more relationship with the output classes. This fact can be clearly seen in Figure 5. Figure 5A shows the distribution of the values of the features obtained from the ApEn criterion in the data space. Figure 5B also shows the same graph for the SampEn criterion. In each of these plots, samples belonging to each class are displayed with a different color and marker. As visually shown in Figure 5B, the SampEn can distinguish the target classes well. However, it is much more difficult to classify samples based on the features produced by the ApEn criterion. According to Figure 5A, the ApEn values of the d_4_ sub-band can partially distinguish the target classes. A higher *F*-statistic for the ApEn of the d_4_ sub-band than the ApEn of the other two sub-bands in Figure 4 confirms this.

The results of feature selection using FSFS algorithm showed that the best feature subset for the proposed method would be 4. In this set, all of SampEn features and the features corresponding to the ApEn of the d_4_ wavelet sub-band are selected. For evaluating the performance of the proposed method, a 10-fold cross-validation experiment was used and during each iteration, 90% of data is used to train the model and the remaining 10% is used to test its performance. In each iteration, new data is used to test the trained model. So, after 10 times repetition of tests, all data is tested during the scenario.

**FIGURE 6:**
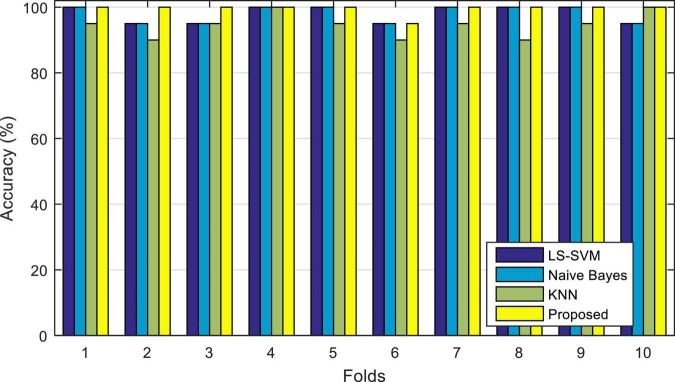
Accuracy of correct diagnosis of the proposed method in comparison with the algorithms used in it.

Figure 6, illustrates the results of the correct diagnosis of the proposed model, during each iteration. In this diagram (and subsequent experiments in this chapter) the accuracy of the proposed method (ensemble model) is compared with each of its learning algorithms (LS-SVM), (KNN), and (NB) used separately.

The results shown in Figure 6 show the percentage of correct diagnosis for each fold of the test. As shown in this figure, the proposed method can improve the accuracy of diagnosing epileptic seizures compared to other compared algorithms. The results of this experiment show that in the case of using the proposed method, the lowest accuracy of correct diagnosis of the epileptic seizures is 95%, the highest accuracy is 100% and the average accuracy is 99.5%. The results related to the accuracy of each of these algorithms are in Table 3.

**TABLE 3:** Summary of prediction results of each tested algorithms.

Title	Average accuracy (%)	Minimum accuracy (%)	Maximum accuracy (%)	Standard deviation
Proposed method	99.5	95	100	1.581
LS-SVM	98	95	100	2.58
KNN	94.5	90	100	3.689
NB	98	95	100	2.582

**FIGURE 7:**
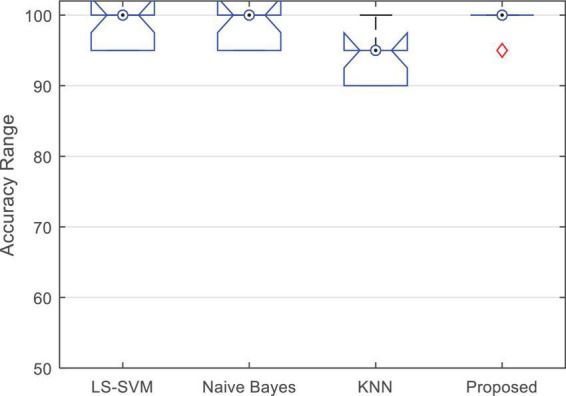
Boxplot of the accuracy changes of different algorithms during 10-fold of experiments.

Figure 7 shows the boxplot of the accuracy changes in each classification algorithm. Based on the results shown in this figure, in addition to the higher median accuracy, the proposed method has higher and closer values for the limits of accuracy changes during different iterations. Figure 7 shows that the proposed method has 95% accuracy in only one iteration (error in classifying one test sample) and in other iterations, it has been able to classify all test samples correctly.

**FIGURE 8:**
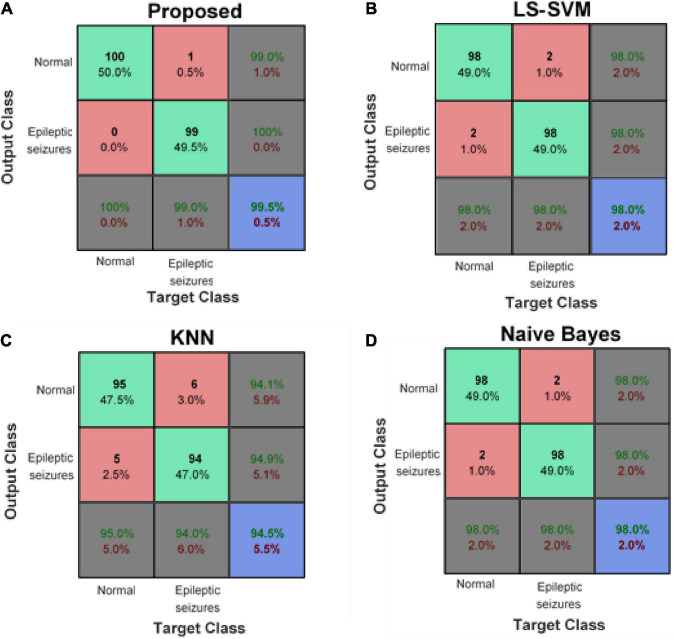
Confusion matrix of panel **(A)** proposed method, **(B)** least squared support vector machine (LS-SVM), **(C)** K-nearest neighbors (KNN), and **(D)** Naive Bayes (NB).

Figure 8 compares the confusion matrix resulting from the detection of epileptic seizures in the proposed model with other learning models. These results show that the proposed method has an average accuracy of 99.5% in classifying healthy and epileptic seizure samples and has misclassified only one of the test samples. However, other compared learning models (despite using the same input features as the proposed method) have a higher error rate. This error reduction in the proposed method is due to the use of ensemble technique. The ensemble-based detection system enables a set of learning models together to cover the error of each of the models used. Figure 8A shows that the proposed method can correctly identify 100% of healthy samples (first column) and 99% of epileptic seizure samples (second column). On the other hand, the confusion matrix of the proposed method shows that 99% of the healthy outputs of the proposed method are correct (the first row of the matrix) and if the proposed method detects the presence of seizures for a sample; this diagnosis is definitely correct (second row of the matrix).

Table 4 summarizes the performance measures obtained from the experiments. In this table, the criteria of sensitivity, specificity, and area under the ROC curve (AUC) are compared. Sensitivity criterion measures the overall proportion of positive class samples (samples with epileptic seizures) that have been correctly classified (Han et al., 2011):


(11) $$Sensitivity=\frac{TP}{TP+FN}$$

**TABLE 4:** Comparison of the efficiency of the proposed method with previous methods.

Algorithm	Accuracy (%)	Sensitivity (%)	Specificity (%)	AUC
Proposed method	**99.50**	**99.01**	**100**	**0.9819**
Chen et al., 2019	99.50	99.08	99.40	0.9814
Wang et al., 2017	99.25	97.98	99.56	0.9637
Jaiswal and Banka, 2017	99.07	98.82	99.32	0.9805
Swami et al., 2016	98.00	96.39	98.57	0.9533
Sarić et al., 2020	95.14	97.56	98.33	0.9384
Hassan et al., 2020	99.00	100	98.00	0.9779

Bold values represent the results related to the proposed diagnosis system.

Where, TP indicates the number of positive class samples that have been correctly identified and FN represents the number of positive class samples that have been identified as negative (healthy). The specificity criterion is used to measure the performance of the classifier in detecting negative samples. This criterion is calculated as follows (Han et al., 2011):


(12) $$Specificity=\frac{TN}{TN+FP}$$

Where, TN is the number of negative class samples that have been correctly identified and FP is the number of negative class samples that have been classified in the positive class, incorrectly. Finally, the AUC criterion is obtained by calculating the area under the ROC curve (true positive rates versus false positive rate values) for each method. This table shows that the proposed method will reduce the FP rate and increase the TP rate, compared to other algorithms. As a result, the proposed algorithm is more efficient in diagnosing epileptic seizures.

All of the methods compared in Table 4, have used the same database. As the results of Table 4 show, the proposed method has better performance than the compared algorithms in terms of the accuracy, specificity, and AUC. Since the proposed method uses a combination of several machine learning methods for disease diagnosis, it is necessary to examine its performance from the aspect of processing time. The experiments executed on a laptop with 64-bit version of Microsoft Windows 10 and an Intel core-i7 processor with frequency of 3.2 GHz and 8 gigabytes of main memory. The processing time measured during three phases of: sample preparation, training, and testing, separately. The total processing time of the sample preparation phase (including: preprocessing, feature extraction, and feature selection steps) was 6.8475 s, which means the proposed method can preprocess and prepare a sample with average processing time of 0.0342 s. The average processing time of the training phase (training all three classifiers with 180 train samples) was 0.0372 s for each iteration. On the other hand, processing time of the phase was only 9.5 ms for each iteration, which means the proposed method was able to diagnose epileptic seizures in each test sample within average processing time of 0.475 ms. Considering the sample preparation time, the class of each new sample will be determined within 0.0342 s. These results indicate that the proposed method is a cost-effective diagnosis system with low computation complexity which can be used in real-world applications.

In the other study by Mouleeshuwarapprabu and Kasthuri (2020), after preprocessing the EEG signal, some features like entropy are extracted and then non-linear vector decomposed neural network (NVDN) is used to classify. In this approach, they did not use feature selection method and the accuracy of this method is 95.60% which is lower than our proposed method.

## 4. Conclusion

Epilepsy is a neural disorder that, if diagnosed early, can prevent injuries to the patient. In this paper, a new method for automatic detection of epileptic seizures using entropy-based features and ensemble learning is presented. In the proposed method, discrete wavelet transform is used to analyze the EEG signal and remove redundant information. The proposed method also uses ApEn and SampEn criteria along with FSFS strategies to describe EEG signals. In this research, it was shown that using the mentioned entropy criteria, an EEG signal can be described through only four features. On the other hand, the use of a combination of several learning models by the ensemble technique in the proposed method, has increased the accuracy of the diagnosis compared to the case where each learning model is used separately. Studies have shown that the proposed ensemble model can be effective in covering the error of the individual learners used in it, and increase the detection accuracy by at least 1.5%. During the experiments, the efficiency of the proposed method in terms of criteria such as accuracy, sensitivity, and specificity were discussed and the results were compared with other learning models. The results showed that the proposed method can diagnose epileptic seizures with an average accuracy of 99.5% and be used as an effective tool in real-world applications.

We had some limitations during our research since recording and collecting a lot of EEG data from epileptic patients is time-consuming and difficult and we had to use provided database. Also, working with EEG signals which are non-stationary and noisy, need much more sensitivity.

In future works, the application of optimization algorithms in selecting the optimal subset of signal features, can be investigated. In the proposed ensemble model, the output of all learning models has the same importance. In some cases, it is necessary to determine the effectiveness of a learner output based on its performance. Therefore, the use of ensemble learning based on weighted voting may be effective in improving the performance of the proposed method.

## Data availability statement

The original contributions presented in this study are included in this article/supplementary material, further inquiries can be directed to the corresponding author.

## Author contributions

MD wrote the manuscript and revised it with the support of ZR. ZR provided the research ideas and guidance. Both authors contributed to the article and approved the submitted version.

## References

[B1] Chen D. Wan S. Xiang J. Bao F. S. (2017). A high-performance seizure detection algorithm based on Discrete Wavelet Transform (DWT) and EEG. *PLoS One* 12:e0173138. 10.1371/journal.pone.0173138 28278203 PMC5344346

[B2] Chen S. Zhang X. Chen L. Yang Z. (2019). Automatic diagnosis of epileptic seizure in electroencephalography signals using nonlinear dynamics features. *IEEE Access* 7 61046–61056.

[B3] Cherian R. Kanaga E. G. (2022). Theoretical and methodological analysis of EEG based seizure detection and prediction: An exhaustive review. *J. Neurosci. Methods* 369:109483. 10.1016/j.jneumeth.2022.109483 35051438

[B4] Daftari C. Shah J. Shah M. (2022). “Detection of epileptic seizure disorder using EEG signals,” in *Artificial intelligence-based brain-computer interface*, eds Bajaj V. Sinha G. R. (Cambridge, MA: Academic Press), 163–188.

[B5] Ebrahimzadeh E. Saharkhiz S. Rajabion L. Oskouei H. B. Seraji M. Fayaz F. (2022). Simultaneous electroencephalography-functional magnetic resonance imaging for assessment of human brain function. *Front. Syst. Neurosci.* 16:934266. 10.3389/fnsys.2022.934266 35966000 PMC9371554

[B6] Ebrahimzadeh E. Shams M. Jounghani A. R. Fayaz F. Mirbagheri M. Hakimi N. (2019). Epilepsy presurgical evaluation of patients with complex source localization by a novel component-based EEG-fMRI approach. *Iran. J. Radiol.* 16:e99134.

[B7] Ebrahimzadeh E. Shams M. Seraji M. Sadjadi S. M. Rajabion L. Soltanian-Zadeh H. (2021b). Localizing epileptic foci using simultaneous EEG-fMRI recording: Template component cross-correlation. *Front. Neurol.* 12:695997. 10.3389/fneur.2021.695997 34867704 PMC8634837

[B8] Ebrahimzadeh E. Shams M. Rahimpour Jounghani A. Fayaz F. Mirbagheri M. Hakimi N. (2021a). Localizing confined epileptic foci in patients with an unclear focus or presumed multifocality using a component-based EEG-fMRI method. *Cogn. Neurodyn.* 15 207–222. 33854640 10.1007/s11571-020-09614-5 PMC7969677

[B9] Gao Y. Gao B. Chen Q. Liu J. Zhang Y. (2022). Deep convolution neural network-based epileptic electroencephalogram (EEG) signal classification. *Front. Neurol.* 11:375. 10.3389/fneur.2020.00375 32528398 PMC7257380

[B10] Han J. Pei J. Kamber M. (2011). *Data mining: Concepts and techniques.* Amsterdam: Elsevier.

[B11] Hassan A. R. Subasi A. Zhang Y. (2020). Epilepsy seizure detection using complete ensemble empirical mode decomposition with adaptive noise. *Knowl. Based Syst.* 191:105333. 10.1016/j.cmpb.2016.08.013 27686704

[B12] Jaiswal A. K. Banka H. (2017). Local pattern transformation based feature extraction techniques for classification of epileptic EEG signals. *Biomed. Signal Process. Control* 34 81–92.

[B13] Jin K. Yan Y. Chen M. Wang J. Pan X. Liu X. (2022). Multimodal deep learning with feature level fusion for identification of choroidal neovascularization activity in age-related macular degeneration. *Acta Ophthalmol.* 100 e512–e520. 10.1111/aos.14928 34159761

[B14] Mouleeshuwarapprabu R. Kasthuri N. (2020). Nonlinear vector decomposed neural network based EEG signal feature extraction and detection of seizure. *Microprocess. Microsyst.* 76:103075.

[B15] Natu M. Bachute M. Gite S. Kotecha K. Vidyarthi A. (2022). Review on epileptic seizure prediction: Machine learning and deep learning approaches. *Comput. Math. Methods Med.* 2022:7751263. 35096136 10.1155/2022/7751263 PMC8794701

[B16] Peterson L. E. (2009). K-nearest neighbor. *Scholarpedia* 4 1883.

[B17] Pisner D. A. Schnyer D. M. (2020). “Support vector machine,” in *Machine learning*, eds Mechelli A. Vieira S. (Cambridge, MA: Academic Press), 101–121.

[B18] Rahimpour A. Pollonini L. Comstock D. Balasubramaniam R. Bortfeld H. (2020). Tracking differential activation of primary and supplementary motor cortex across timing tasks: An fNIRS validation study. *J. Neurosci. Methods* 341:108790. 10.1016/j.jneumeth.2020.108790 32442439 PMC7359891

[B19] Ranjbari S. Khatibi T. Vosough Dizaji A. Sajadi H. Totonchi M. Ghaffari F. (2021). CNFE-SE: A novel approach combining complex network-based feature engineering and stacked ensemble to predict the success of intrauterine insemination and ranking the features. *BMC Med. Inform. Decis. Mak.* 21:1. 10.1186/s12911-020-01362-0 33388057 PMC7778826

[B20] Richman J. S. Lake D. E. Moorman J. R. (2004). Sample entropy. *Methods Enzymol*. 384 172–184. 15081687 10.1016/S0076-6879(04)84011-4

[B21] Sadjadi S. M. Ebrahimzadeh E. Soltanian-Zadeh H. (2022). “fMRI functional connectivity analysis for localizing epileptic focus,” in *Proceedings of the 2022 30th international conference on electrical engineering (ICEE)*, Tehran.

[B22] Sadjadi S. M. Ebrahimzadeh E. Shams M. Seraji M. Soltanian-Zadeh H. (2021). Localization of epileptic foci based on simultaneous EEG–fMRI data. *Front. Neurol.* 12:645594. 10.3389/fneur.2021.645594 33986718 PMC8110922

[B23] Sarić R. Jokić D. Beganović N. Pokvić L. G. Badnjević A. (2020). FPGA-based real-time epileptic seizure classification using Artificial Neural Network. *Biomed. Signal Process. Control* 62:102106.

[B24] Sharma R. Pachori R. B. Sircar P. (2020). Seizures classification based on higher order statistics and deep neural network. *Biomed. Signal Process. Control* 59:101921. 10.1186/s12868-016-0283-6 27534393 PMC5001212

[B25] Shoeibi A. Ghassemi N. Khodatars M. Moridian P. Alizadehsani R. Zare A. (2021). Detection of Epileptic Seizures on EEG Signals Using ANFIS Classifier, Autoencoders and Fuzzy Entropies. *Biomed. Signal Process. Control* 73:103417.

[B26] Siddiqui M. K. Morales-Menendez R. Huang X. Hussain N. (2020). A review of epileptic seizure detection using machine learning classifiers. *Brain Inform.* 7:5. 32451639 10.1186/s40708-020-00105-1 PMC7248143

[B27] Singh K. Malhotra J. (2022). Smart neurocare approach for detection of epileptic seizures using deep learning based temporal analysis of EEG patterns. *Multimed. Tools Appl.* 81 29555–29586.

[B28] Srinivasan V. Eswaran C. Sriraam N. (2007). Approximate entropy-based epileptic EEG detection using artificial neural networks. *IEEE Trans. Inform. Technol. Biomed.* 11 288–295. 10.1109/titb.2006.884369 17521078

[B29] Swami P. Gandhi T. K. Panigrahi B. K. Tripathi M. Anand S. (2016). A novel robust diagnostic model to detect seizures in electroencephalography. *Expert Syst. Appl.* 56 116–130.

[B30] University of Bonn (2021). *EEG database from university of Bonn*. Available Online at: https://www.ukbonn.de/en/epileptology/workgroups/lehnertz-workgroup-neurophysics/downloads/ (accessed November 2021).

[B31] Vaghela V. B. Vandra K. H. Modi N. K. (2014). Entropy based feature selection for multi-relational Naïve Bayesian classifier. *J. Int. Tech. Inf. Manag.* 23:2.

[B32] Wang L. Xue W. Li Y. Luo M. Huang J. Cui W. (2017). Automatic epileptic seizure detection in EEG signals using multi-domain feature extraction and nonlinear analysis. *Entropy* 19:222. 10.1109/TNSRE.2020.2973434 32078551

[B33] Yang B. Xu S. Chen H. Zheng W. Liu C. (2022). Reconstruct dynamic soft-tissue with stereo endoscope based on a single-layer network. *IEEE Trans. Image Process.* 31 5828–5840. 10.1109/TIP.2022.3202367 36054398

[B34] Yang F. J. (2018). “An implementation of Naive Bayes classifier,” in *Proceedings of the 2018 international conference on computational science and computational intelligence (CSCI)*, (Las Vegas, NV: IEEE), 301–306.

[B35] Zhou M. Tian C. Cao R. Wang B. Niu Y. Hu T. (2018). Epileptic seizure detection based on EEG signals and CNN. *Front. Neuroinform.* 12:95. 10.3389/fninf.2018.00095 30618700 PMC6295451

